# Deep learning framework for interpretable quality control of echocardiography video

**DOI:** 10.1002/mp.17722

**Published:** 2025-03-04

**Authors:** Liwei Du, Wufeng Xue, Zhanru Qi, Zhongqing Shi, Guanjun Guo, Xin Yang, Dong Ni, Jing Yao

**Affiliations:** ^1^ School of Biomedical Engineering, Shenzhen University Medical School Shenzhen University Shenzhen China; ^2^ Department of Ultrasound Medicine at the Affiliated Hospital of Medical School Nanjing University Nanjing China; ^3^ Medical Imaging Centre, Affiliated Hospital of Medical School Nanjing University Nanjing China; ^4^ Yizheng Hospital of Nanjing Drum Tower Hospital Group Yangzhou China

**Keywords:** echocardiography video, multitask network, quality control, real‐time, visualized explanation

## Abstract

**Background:**

Echocardiography (echo) has become an indispensable tool in modern cardiology, offering real‐time imaging that helps clinicians evaluate heart function and identify abnormalities. Despite these advantages, the acquisition of high‐quality echo is time‐consuming, labor‐intensive, and highly subjective.

**Purpose:**

The objective of this study is to introduce a comprehensive system for the automated quality control (QC) of echo videos. This system focuses on real‐time monitoring of key imaging parameters, reducing the variability associated with manual QC processes.

**Methods:**

Our multitask network analyzes cardiac cycle integrity, anatomical structures (AS), depth, cardiac axis angle (CAA), and gain. The network consists of a shared convolutional neural network (CNN) backbone for spatial feature extraction, along with three additional modules: (1) a bidirectional long short‐term memory (Bi‐LSTM) phase analysis (PA) module for detecting cardiac cycles and QC targets; (2) an oriented object detection head for AS analysis and depth/CAA quantification; and (3) a classification head for gain analysis. The model was trained and tested on a dataset of 1331 echo videos. Through model inference, a comprehensive score is generated, offering easily interpretable insights.

**Results:**

The model achieved a mean average precision of 0.962 for AS detection, with PA yielding average frame errors of 1.603±1.181 (end‐diastolic) and 1.681±1.332 (end‐systolic). The gain classification model demonstrated robust performance (Area Under the Curve > 0.98), and the overall processing speed reached 112.4 frames per second. On 203 randomly collected echo videos, the model achieved a kappa coefficient of 0.79 for rating consistency compared to expert evaluations

**Conclusions:**

Given the model's performance on the clinical dataset and its consistency with expert evaluations, our results indicate that the model not only delivers real‐time, interpretable quality scores but also demonstrates strong clinical reliability.

## 1 INTRODUCTION

Echocardiography (echo) video has gained widespread acceptance in the diagnosis of clinical heart diseases due to its noninvasive, cost‐effective, portable, and accessible imaging approach.^1^ Echo video can present abundant temporal and spatial information about patient's heart, which can aid physicians in assessing the structure and function of the heart, such as detecting congenital heart disease and measuring the ejection fraction (EF). However, the foundation for accurate diagnosis lies in obtaining high quality echo video. Quality control (QC) is therefore essential in reducing misdiagnosis.

The importance of QC has been elucidated,^2^, ^3^ and guidelines on how to perform QC have been provided.^4^, ^5^ While these methods have proven to be effective, their clinical implementation is impractical due to the interobserver and intra‐observer variability, as well as the labor‐intensive and time‐consuming nature of the process. Additionally, in clinical, to become an experienced operator requires extensive practice and expert guidance,^6^ which will significantly burden clinical work. With the development of deep learning, AI‐assisted scanning^7^ has the potential to alleviate this issue by assisting novice operators quickly obtaining standard views. However, the key step lies in how to explain the indication from artificial intelligence (AI) to people and gain their trust in its capabilities. Therefore, a fully automated and interpretable echo video QC system is highly demanded.

The QC framework for echo video usually consists of a protocol for the target planes^8^ specified under expert guidance, along with networks for quantitatively evaluating items in the protocol and the selection of QC target. The protocol generally includes QC criteria (as shown in Table 1) that includes all the elements constituting a standard data, as well as their corresponding weight scores representing the importance of each criterion. When referring to criteria, it is worth noting that in clinical practice, effective diagnosis often requires data covering one or more complete cardiac cycles. Therefore, ensuring the presence of a complete cardiac cycle in the echo video is a prerequisite for QC, and the process should be conducted on individual cardiac cycles, a step overlooked in previous studies. Additionally, in echo cine, the chambers and structures of the heart undergo continuous changes throughout the cardiac cycle. However, they remain relatively stable during the isovolumetric contraction and relaxation phases. Therefore, we have selected end‐diastole (ED) and end‐systole (ES) as the targets for QC. In summary, the general QC process for echo videos of a specific plane should contain the following steps: Firstly, determine if the ultrasound video contains complete cardiac cycles. Then, use these complete cardiac cycles as fundamental units, identifying the key frames within each individual cardiac cycle as QC targets. Finally, conduct QC using these targets and obtain the final score and insights.

**TABLE 1:** QC protocol of A4C.

Item	Criteria	Score
Complete cardiac cycle	Sequence from ED to ES and back to ED, or from ES to ED and back to ES. Others	Start QC End QC with 0
Gain	High gain or low gain. Others	0 +1
Depth	The complete chest silhouette covers 2/3 to 4/5 of the entire fan‐shaped area. Others	+1 0
Cardiac axis angle	Within −20 to 20 degrees. Others	+1 0
Anatomical structures	Key structures in both the ED frame and ES frame are clearly visible. Others	+2 0

Abbreviations: A4C, apical 4 chamber; QC, quality control.

**FIGURE 1:**
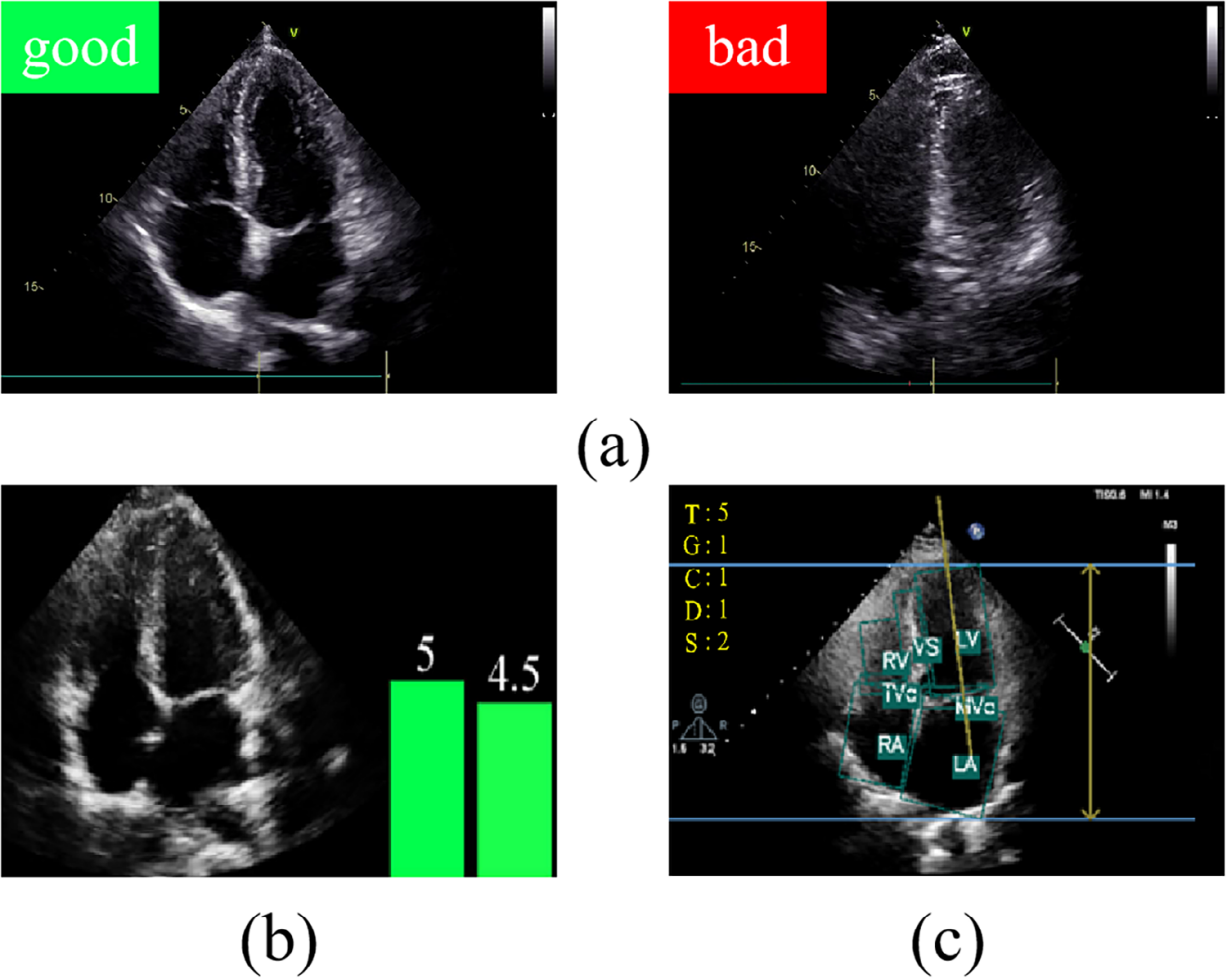
Comparison of outcomes between (a) QC, (b) QC, and (c) QC. QC, quality control.

Recently, there have been significant advancements in the application of AI in echo, for example, in view classification^9^, ^10^ and cardiac function measurement.^11^, ^12^ However, there is few research in the field of echo quality analysis. As illustrated in Figure 1, studies in this field are generally categorized into three types:

**Quality classification**: This type roughly classifies the quality of an echo image as either good or bad. Zamzmi et al.^13^ utilized a classification head contains convolutional layers and fully connected layers to classify the quality of an echo image as either good or bad. Its main limitation lies in the lack of interpretability, as the model simply categorize images into good and bad without providing any specific indicators or information

**Quality assessment (QA)**: This involves scoring echo images or videos based on criteria provided by experts. Abdi et al.^14^ employed Particle Swarm Optimization to train a regression model based on a customized deep convolutional neural network (CNN) for automated QA in apical 4 chamber (A4C) view. In another work, Abdi et al.^15^ developed a regression model based on CNN for spatial feature extraction and five recurrent neural networks (RNN) for temporal feature extraction to regress the quality scores of five different views. Similar models for QA have also been explored.^16^, ^17^ Comparable to quality classification, QA methods offer interpretability to some extent, the assigned scores align with specific criteria set by experts. However, for novices, the results may still be difficult to effectively guide them in taking the next steps to obtain high‐quality data.

**QC**: In addition to scoring, QC exhibits excellent interpretability, allowing operators to directly obtain explanations from the resulting images and assisting them in optimizing performance. Dong et al.^18^ proposed a CNN‐based real‐time QC framework for fetal A4C views by evaluating important echo imaging parameters (e.g., gain and zoom). The framework consists of three different networks: a basic CNN to classify the A4C views in the raw echo image; a deeper CNN to determine the gain and zoom of the echo image; and an aggregated residual visual block net to detect the anatomical structures (ASs) on the image. Nevertheless, this work only takes into account a single image but ignores the periodicity of the heartbeat. What's more, this framework is designed in a cascade manner, which may appear cumbersome and could potentially lead to error accumulation. Consequently, its clinical applicability and scalability are somewhat limited.

Based on the above discussion, a more concise, efficient, and comprehensive QC system for echo video is highly desirable. To the best of our knowledge, this is the first and most comprehensive study of QC for echo video in which an end‐to‐end fully automated QC system for echo video was developed with strong interpretability and real‐time performance. Drawing from expert clinical experience, five key aspects were taken into consideration when conducting QC: completeness of cardiac cycle, gain, depth, cardiac axis angle (CAA), and presence of key structures. To assess these aspects in an echo video, a lightweight multitask network comprising three modules was designed. These modules include a phase analysis (PA) module, a structure analysis module, and a gain classification (GC) module. For an input echo video, it first goes through backbone to extract features for each frame. Then, a PA module based on bidirectional long short‐term memory (Bi‐LSTM) analyzes the completeness of the cardiac cycle and determines QC targets. Following this, the structure analysis module employs an oriented object detector for structure detection and quantitative analysis of CAA and depth through postprocessing of the detection results. Finally, a GC module is utilized for gain analysis. Based on the outputs from the system, an echo video can be quantified as either a standard view (if a total score of 5 is obtained) or others. These quantitative scores provide operators with valuable insights into image quality, allowing them to make informed decisions about further actions or adjustments.

The specific contributions of this article can be summarized as follows:

1. An automatic echo QC system with strong interpretability has been developed, providing a comprehensive evaluation score for echo videos based on analysis of five key aspects: completeness of the cardiac cycle, gain, depth, CAA, and structural integrity, with real‐time performance. To our best knowledge, this represents the first and most comprehensive QC system implemented for echo videos.
2. In the cardiac cycle detection module, we designed a concise and efficient function to construct the left ventricular volume curve (LVVC) and use it as ground truth (GT) for the regression network based on expert annotations. With the trained module, we are able to detect key frames and assess the completeness of the cardiac cycle, as well as obtain QC targets based on the results.
3. In the structure analysis module, we integrated a postprocessing module that can analyze depth and CAA, making effective use of the network output information and avoiding the need for additional model design.
4. We conducted human–machine comparative experiments on a large dataset of actual clinical data, demonstrating the advantages and feasibility of utilizing the proposed approach in clinical practice, achieving a kappa coefficient of 0.79.

## 2 MATERIALS

In this section, we provide a detailed description of the QC protocol and the datasets used. The view we utilized is the A4C view, as it is one of the most informative yet challenging views.

### 2.1 QC protocol

**FIGURE 2:**
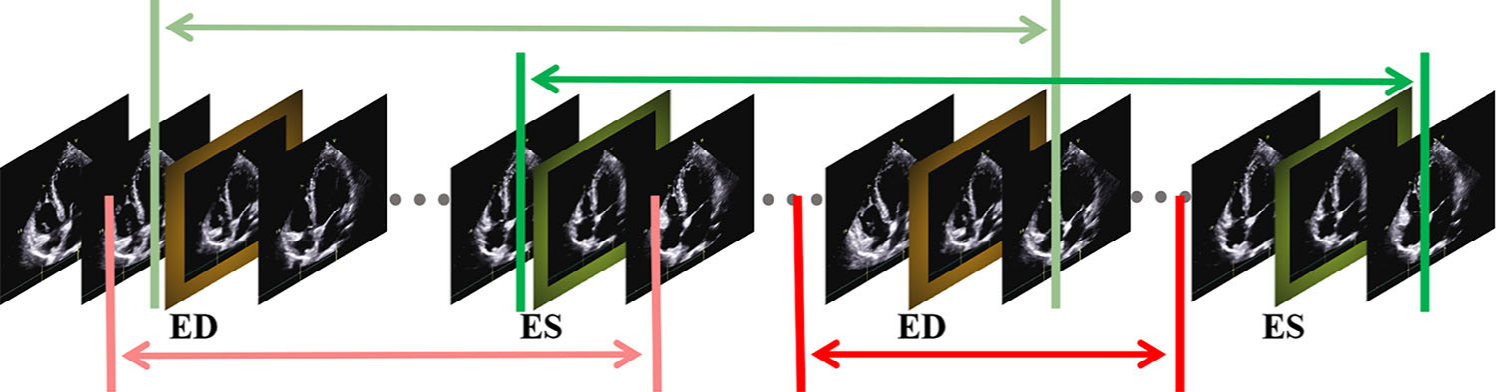
Illustration of cardiac cycle completeness: The green ranges indicate a complete cardiac cycle, while the red ranges indicate an incomplete cardiac cycle.

Two experts from Nanjing Drum Tower Hospital formulated the protocol by drawing on guidelines and combining them with clinical practice as shown in Table 1. All these metrics are defined on the ES frame. Specifically, in order to enhance reliability, for AS detection, both the ED frame and ES frame within one cardiac cycle need to be analyzed. We set the score for a qualified echo video at 5, starting from 0, and the score is increased based on the following criteria:

**Complete cardiac cycle**: As discussed earlier, whether the video contains a complete cardiac cycle is a prerequisite of the diagnostic significance of the video. As shown in Figure 2, we define a complete cardiac cycle as one that includes two ES (ED) frames in a cardiac cine, with one ED (ES) frame contained between these two frames (i.e., ED‐ES‐ED or ES‐ED‐ES). Due to its role as a prerequisite for diagnosis, if the cardiac cycle is incomplete, we directly assign a total score of 0.

**FIGURE 3:**
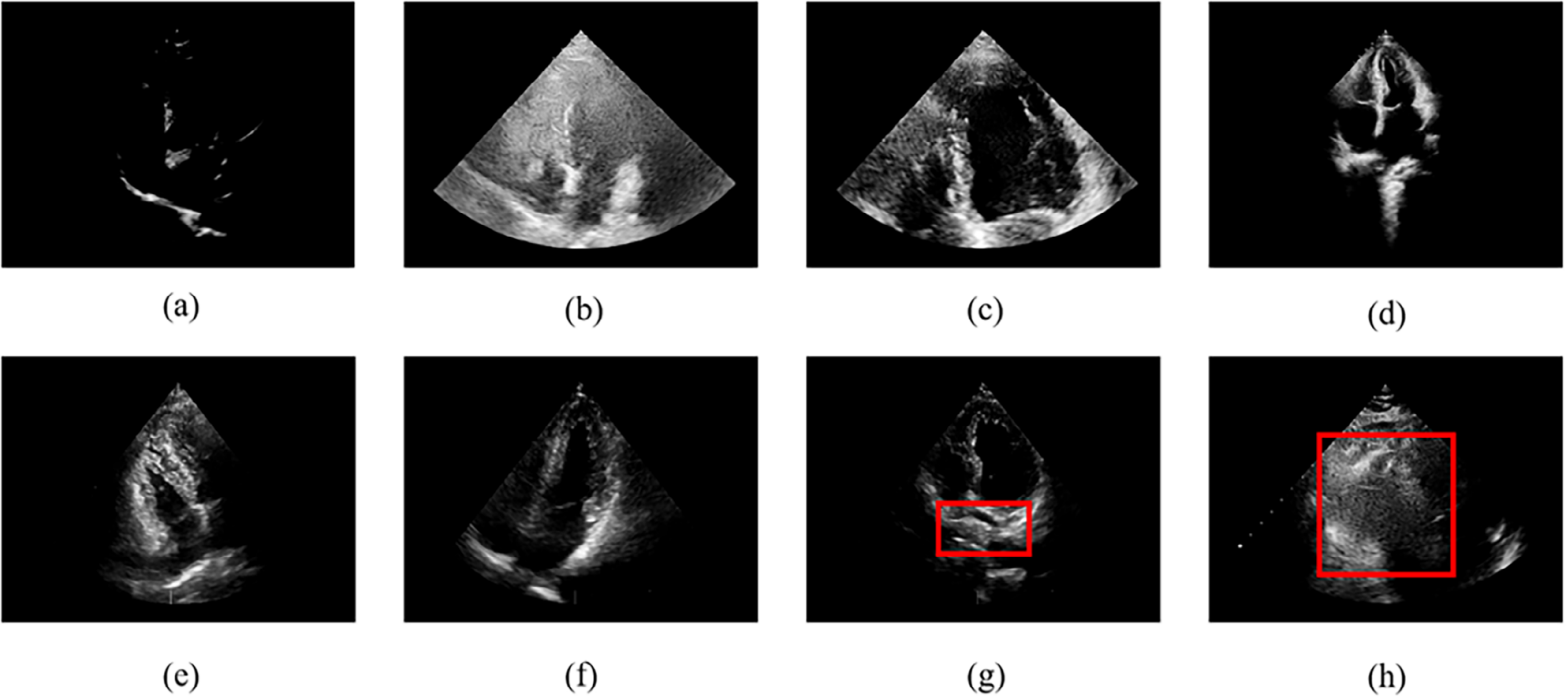
Display of some examples of nonstandard echo: gain issues (a, b), depth issues (c, d), CAA issues (e, f), structural issues (g, h). CAA, cardiac axis angle.

**Gain**: Gain can affect the brightness and clarity of the image. Excessive gain may lead to blurred boundaries and increased noise, while insufficient gain can result in poor contrast and some structures being invisible as indicated in Figure 3a,b. If the gain is appropriate, then score plus one; otherwise, zero.

**Depth**: The scanning depth determines the proportion of the heart in the scanning area. If the depth is too shallow, the imaging proportion will increase, making it unable to display all structures (Figure 3c); if it is too deep, the frame rate will be lost, and the imaging proportion will decrease (Figure 3d). When the complete chest silhouette covers 2/3 to 4/5 of the entire fan‐shaped area, then score plus one; otherwise, zero.

**CAA**: In A4C view, the cardiac axis is defined as the line connecting the midpoint of the mitral valve annulus to the cardiac apex. The CAA is the angle between the cardiac axis and the perpendicular line of the screen. If the cardiac axis deviates, it can cause missing structures in the sector such as Figure 3e,f. When the CAA is within −20 to 20 degrees, then score plus one; otherwise, zero.

**ASs**: In a high‐quality A4C view echo image, seven ASs should be clearly visible: the left and right ventricles, left and right atria, mitral and tricuspid valves, and the interventricular septum. Except for the mitral and tricuspid valves, all other structures must be visible due to individual variability. When conducting structural detection on a cardiac cine representing one complete cycle, both the ED and ES frames require analysis. If those essential structures, excluding the mitral and tricuspid valves, are clearly visible in both frames, then score plus two; otherwise (e.g., as shown in Figure 3g,h), zero.

### 2.2 Dataset

All data in this study were sourced from Nanjing Drum Tower Hospital and divided into three nonoverlapping datasets: the phase analysis (PA) dataset, anatomical structure (AS) dataset, and gain classification (GC) dataset. These datasets were utilized for training and testing the PA module, key structure detection module, and gain module, respectively. The data were collected from December 2021 to November 2023 using General Electric (GE) Vivid E9, E95; Philips iE Elite, EPIQ 5C, EPIQ 7c, and Philips EPIQ CVx ultrasound system (CVX) ultrasound machines, resulting in a total of 1331 videos (per patient). Each video contains at least one cardiac cycle. Due to variations in the acquisition devices, some data have a resolution of 800× 600, while others have a resolution of 1016 ×708. Detailed information for each dataset is provided below.

For AS dataset. It consists of 331 echo videos. To enhance the performance of the oriented object detection module, among these videos, 169 exhibit an abnormal cardiac axis, while the remaining samples represent normal cases. For each video, the ED and ES frames were selected, and rotation bounding box annotations were provided for the left atrium, left ventricle, right atrium, right ventricle, interventricular septum, mitral valve, and tricuspid valve. We used 80％ of the data for the training set, 20％ for the testing set.

The PA dataset consists of 730 echo videos without electrocardiogram (ECG) information. During annotation, within each video, cardiac cycles are selected, and the positions of the ED frame and ES frame (three frames in a single cardiac cycle are labeled, such as ED‐ES‐ED or ES‐ED‐ES) within that cycle are labeled. Based on these labels, a LVVC is generated as GT during the training phase using a function we designed. The data partitioning method aligns with that of the AS dataset.

The gain classification (GC) dataset consists of heart ultrasound images from 273 videos, which are labeled as high gain, medium gain, and low gain. Among these, 102 videos have high gain, 99 videos have medium gain, and 72 videos have low gain. For these videos, we partition the data into an 80% training set and a 20% testing set (based on cases). From each video, 50% of the frames are randomly selected, forming the final GC dataset, which includes 13 880 images. The training set contains 10 943 images, and the testing set contains 2937 images

## 3 METHOD

### 3.1 Overall framework and workflow

**FIGURE 4:**
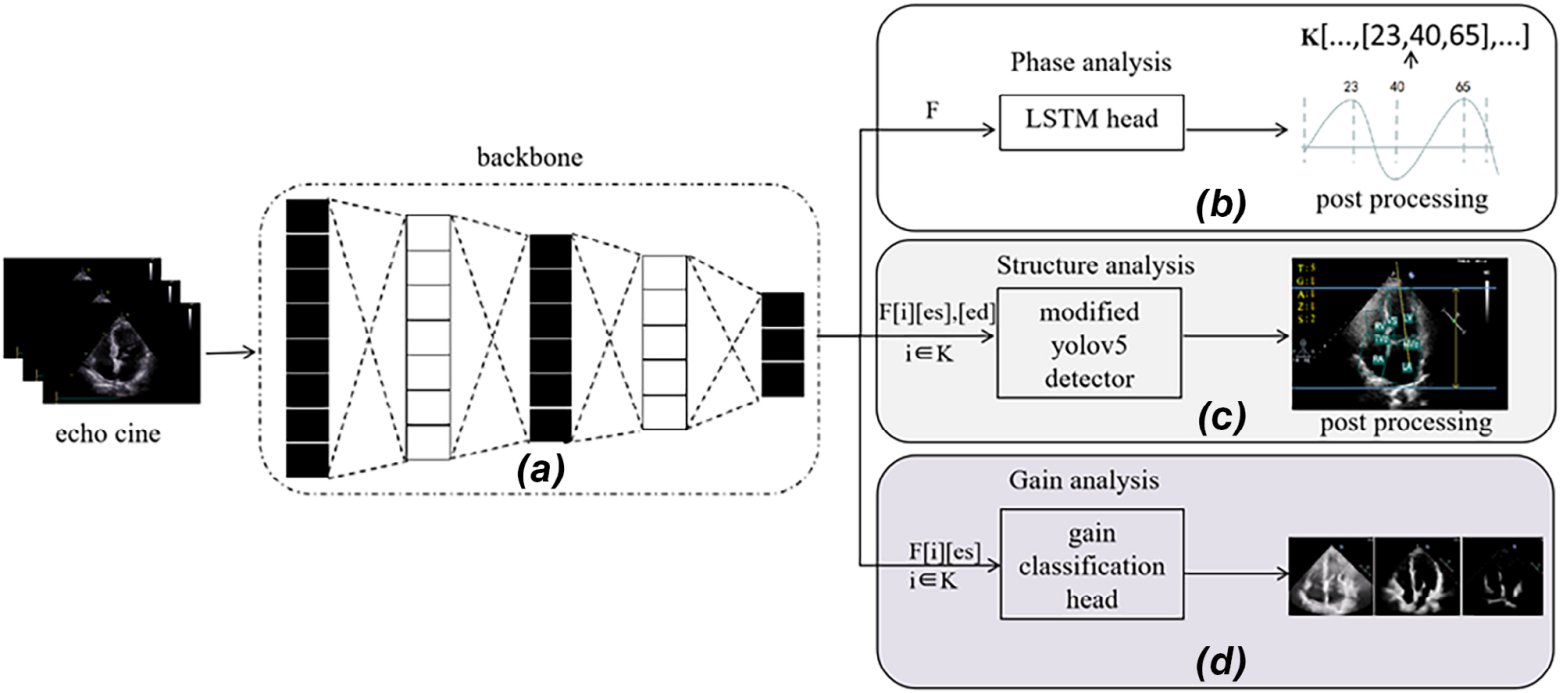
Proposed framework for echo video QC. (a)backbone with shared parameters for feature extraction, (b) keyframe detection module, (c) Rotation box‐based detection module, and(d) GC module. F refers to the features extracted from backbone. F[i][ed][es] specifically denotes the ED, ES frame of the *i*th cardiac cycle. ED, end‐diastole; ES, end‐systole; GC, gain classification; QC, quality control.

**FIGURE 5:**
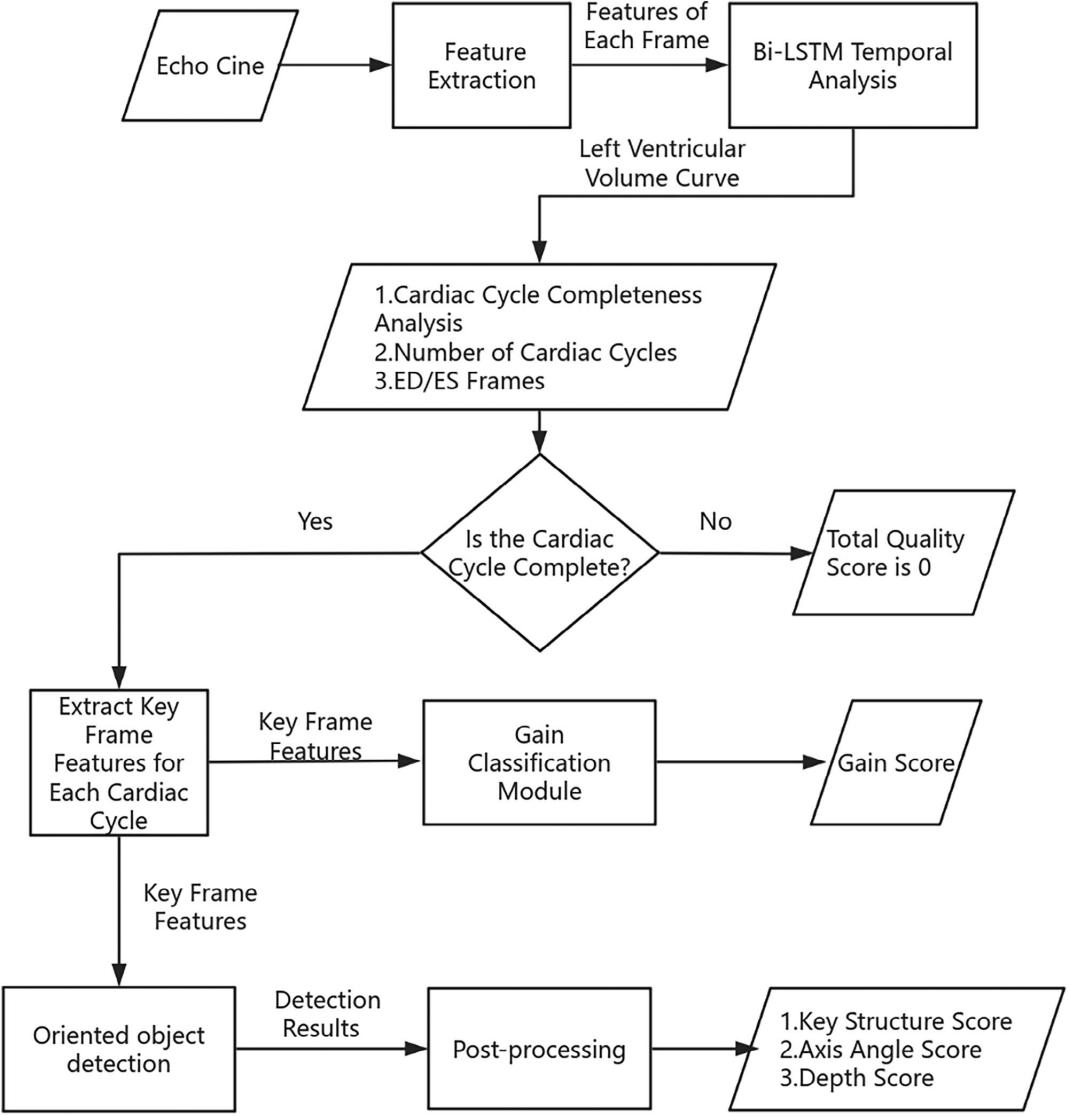
A complete cardiac cycle is a prerequisite for initiating QC, followed by the evaluation of various QC metrics through two additional modules. QC, quality control.

In this study, we propose a generic multitask QC framework for echo videos, comprising a phase assessment module for evaluating the completeness of cardiac cycles and acquiring QC targets, a structure detection module capable of identifying key structures and analyzing depth and CAA, and a gain evaluation module for analyzing gain. Figure 4 presents the main components of the proposed framework and illustrates this process. Notably, our system possesses a real‐time capability and facilitates comprehensive QC tailored specifically for echo video. The QC process begins by analyzing the completeness of the echo video's cardiac cycles. If they are complete, for each cardiac cycle, we select the ES and ED frames as the targets for QC. Through the structure analysis module and GC module, we can obtain scores corresponding to the criteria listed in the Table 1. Figure 5 illustrates the workflow of the entire QC process, showing the steps taken from the initial video analysis to the final quality evaluation.

### 3.2 Shared backbone for feature extraction

In this study, we utilize the YOLOv5^19^ backbone as the shared feature extractor for our multitask framework, which enables effective and efficient processing of echo videos. YOLOv5, known for its exceptional balance between accuracy, speed, and computational efficiency, has been widely demonstrated in various computer vision tasks. Compared to other well‐established backbones, such as ResNet or visual geometry group (VGG), YOLOv5 offers several advantages, particularly in real‐time applications. The lightweight nature of YOLOv5, especially the YOLOv5n version, ensures that the model can operate efficiently even with limited computational resources, making it suitable for clinical environments where rapid analysis is required.

Additionally, by integrating the three modules (PA, Structure Analysis, and GC) into a shared YOLOv5 backbone, we streamline the entire process. This unified architecture enables the efficient reuse of features across all tasks, eliminating the need for redundant computations commonly seen in traditional sequential or independent task pipelines. By leveraging the same extracted features for each module, our framework not only reduces computational overhead but also ensures consistent high performance across multiple tasks. This streamlined multitask approach enhances the robustness and scalability of our solution, making it highly suitable for echo image analysis in real‐time clinical settings.

### 3.3 PA

The purpose of PA has two aspects. Firstly, its objective is to ascertain whether the current echo video includes a complete cardiac cycle (a prerequisite for QC). Subsequently, it aims to select targets for QC, namely the end‐diastole (ED) and V (ES) frames. The assessment of cardiac cycle completeness can be conducted through the analysis of key frames. A complete cardiac cycle should consist of the sequence from ED to ES and back to ED, or from ES to ED and back to ES.

The recent advancements in the analysis of echo phase have shown that regression modeling of LVVC is an effective approach. Taheri Dezaki et al.^20^ utilized a combination of CNN‐based spatial feature extraction module and RNNs to model temporal dependencies that was employed for regression of LVVC.

In this module, we have devised a concise and efficient function to simulate the variation of left ventricular volume over one cardiac cycle. The function curve serves as the GT for training this regression module. Additionally, we utilize Bi‐LSTM^21^ to extract temporal features from the spatial feature sequences extracted from the backbone and perform regression on the LVVC. The function is as follows:

(1) $$y_{t}=\left\{\begin{array}{c} sin\left(\frac{3\pi }{2}-\frac{\pi \left(f_{es}-x\right)}{\left(f_{ed}-f_{es}\right)}\right)\;if\;x\in DP\\ \sin\left(\frac{3\pi }{2}+\frac{\pi \left(x-f_{es}\right)}{\left(f_{es}-f_{ed}\right)}\right)\;if\;x\in SP \end{array}\right.$$

**FIGURE 6:**
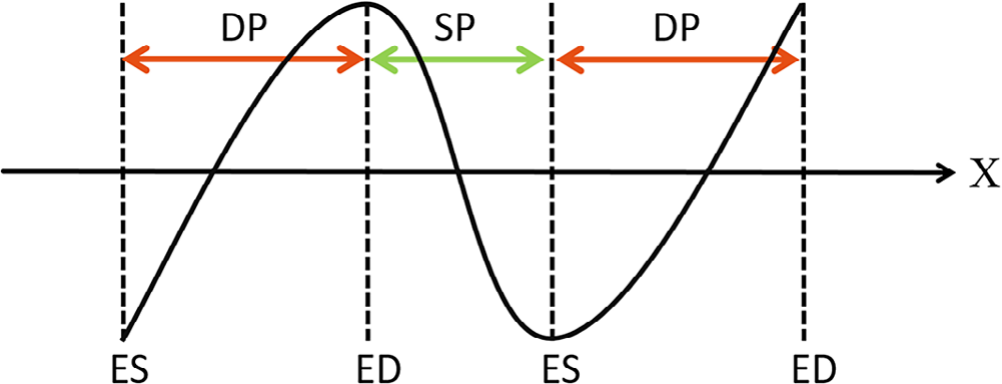
An example of a generated LVVC.

Here $y_{t}$ represents the GT corresponding to time *t* (the *t*th frame), x represents the position of the current frame in the echo video. diastole phase (DP) and systole phase (SP) respectively refer to the diastole phase and systole phase. $f_{ed}$ and $f_{es}\;$ indicate the positions of the ED and ES frames, respectively, that form the current phase (DP or SP). The LVVC construction is illustrated in Figure 6.

**FIGURE 7:**
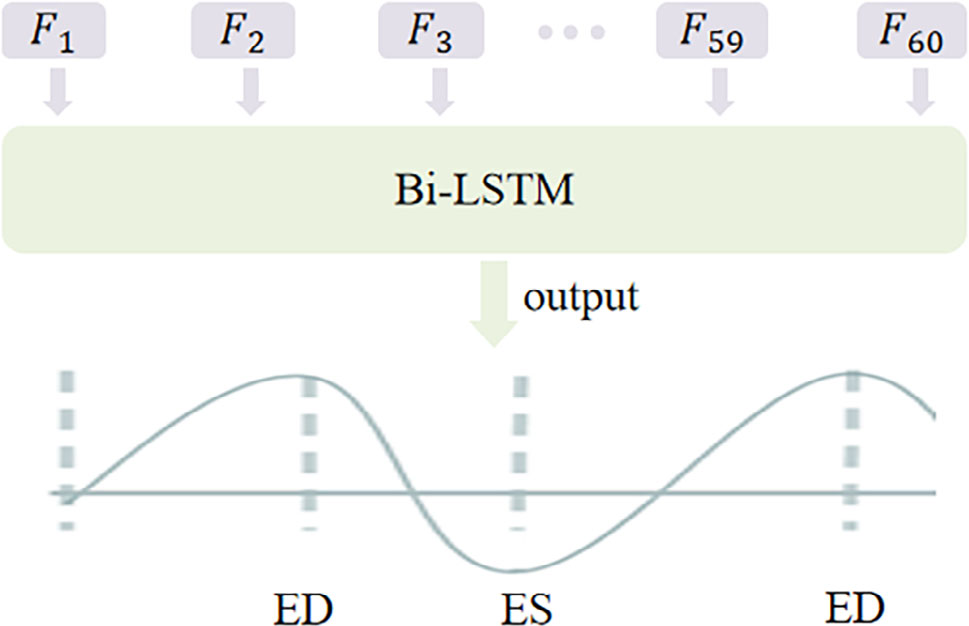
For the obtained regression curve, we consider the peak as the ED frame and the trough as the ES frame. ED, end‐diastole; ES, end‐systole.

During the training process, each video randomly selected a segment containing 60 frames. This length exceeds that of an average cardiac cycle in our dataset, which is 46 frames. Importantly, this selection process ensures that the three labeled frames are included within the 60‐frame range. As a result, at least one complete cardiac cycle is guaranteed to be included in the training data. Through annotating the video segments, we can obtain the corresponding GT using function 1. Subsequently, we input these 60 frames of video segments into the model. Using CNN, we can extract spatial feature set F. Bi‐LSTM then extracts spatiotemporal features from F and trains our Bi‐LSTM based on the GT. The postprocessing for identifying key frames is illustrated in Figure 7.

### 3.4 Structure analysis

This section consists of an oriented object detection head and a postprocessing module. Due to the rich information provided by the oriented object detection structure, including the orientation, position, and size of objects, we can directly utilize these details to evaluate CAA and depth, thus avoiding the need for additional data labeling and model design. The following is a detailed introduction:

**Oriented object detection**: The rotation detector provides orientation and scale information, aiding in a better understanding of the target's state. Several advanced rotation detectors^22^, ^23^, ^24^ have been introduced, with angle regression serving as their foundation. However, these methods encounter the challenge of discontinuous boundaries. Yang et al.^25^ tackled this issue by employing circular smooth labels, treating the prediction of object angles as a classification problem. This approach can be easily extended to other object detection networks. In this paper, we employed a modified rotation detector by integrating the techniques proposed by Yang et al.^25^ with YOLOv5, thereby enhancing its real‐time capabilities.

**FIGURE 8:**
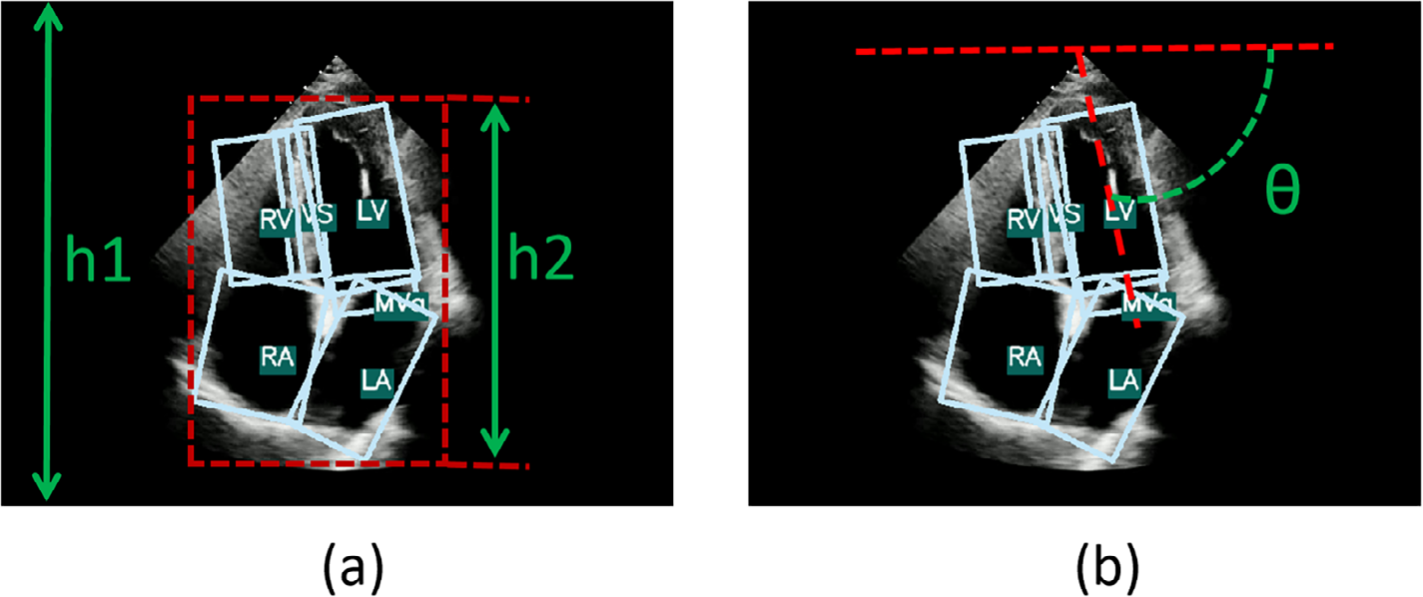
Approximate representation of depth and CAA, D=h2/h1,C=θ∈[0,π]. CAA, cardiac axis angle.

**Postprocessing**: The results of the rotation box object detection provide rich information, including size and orientation, which are closely related to the depth and CAA in our QC indicators. Specifically, for depth, its features in the image are represented by the size of various structures and the entire heart's imaging. Therefore, we can approximate depth by calculating the ratio of the maximum bounding box height(h2) of all structures to the height of the entire image(h1) and use it as parameter **D**, as shown in Figure 8a. As for the CAA, it can be approximated by calculating the angle between the major axis of the left ventricle detection box and the *X*‐axis and denote it as parameter **C** (as shown in Figure 8b. In the subsequent quantification, we will use these two parameters to represent depth and CAA respectively.

To obtain quantitative results for these two parameters, we assume that the **D** and **C** of data collected by doctors follow a normal distribution. Based on this assumption, we conducted statistical analysis on 162 normal data collected by doctors to obtain their standard deviations and means: **D** (mean = 0.6183, std = 0.15) and **C** (mean = 84.5, std = 15). Then, we established two threshold ranges based on experience:(0.5, 0.75) and (75°, 95°) for depth and CAA. Values within the threshold range are considered normal, while values outside the threshold range are considered abnormal.

### 3.5 GC

To the criterion of gain, we design a simple classification head to determine the level of gain. Here, we compare two paths: one extracts shallow features from the shallow convolution of the backbone (considering that image brightness is a low‐level feature), while the other extracts deep features from the last layer. We compare the performance of both features after classification through fully connected layers. The results show no significant difference between the two. Considering network consistency, we choose to input the deep features into the fully connected layers.

### 3.6 Training process

Considering that the object detection model requires higher sensitivity to detailed features compared to the other two modules, we first trained the oriented object detection model. After completing this training, we used the feature extractor (backbone) of the oriented object detection model as a shared feature extractor. During the training process, for the other two modules, such as the features before inputting them into the Bi‐LSTM pooling layer and the features before inputting them into the GC linear layer, these features are extracted by this shared feature extractor. Additionally, no further updates are made to the backbone parameters, and only the parameters of the Bi‐LSTM and classifier are updated, during the training of the Bi‐LSTM, the length of the training data is fixed at 60 frames.

## 4 EXPERIMENTS AND RESULTS

### 4.1 Experimental setup

The data partitioning has been discussed in the dataset section above. Three datasets are used for training three distinct tasks. All experiments were conducted on a Linux system with an Intel(R) Xeon(R) Silver 4210R CPU and an NVIDIA A6000 GPU. The final model demonstration occurred on a Windows system using an NVIDIA RTX 3060 GPU and 11th Gen Intel(R) Core (TM) i7‐11700 CPU.

### 4.2 Experiment for resolution

The resolution of the data significantly impacts model performance. Particularly for our system's rotation detection, detecting small structures becomes challenging at lower resolutions. While higher resolutions offer more detailed information, they also entail increased storage and computational burdens. This is particularly noticeable in video data, where a single video often contains multiple frames. Therefore, in this section, our aim is to find a good balance between model performance and computational cost through experiments.

We conducted a series of experiments to explore the effects of different resolutions (256 × 256, 416 × 416, 512 × 512, and 1024 × 1024) on rotation detector performance and computational memory consumption (MC). Additionally, we evaluated the overall performance of the model's scoring system by analyzing its consistency with expert group ratings using the Cohen's Kappa coefficient. Here, we uniformly use 60 frames of video data. The experimental results are presented in Tables 2 and 3.

**TABLE 2:** Impact of different resolutions on the performance of the detection module.

Resolution	MAP	MP	MR	MC(MB)
256×256	0.865	0.873	0.897	86.69
416×416	0.936	0.935	0.944	202.68
512×512	**0.966**	**0.964**	**0.962**	290.68
1024 ×1024	0.951	0.956	0.952	1098.68

Bold values indicate the best performance in each category.

Abbreviations: MC, memory consumption; MP, mean precision; MR, mean recall.

**TABLE 3:** Impact of different resolutions on the performance of the scoring system.

Resolution	256×256	416 ×416	512 ×512
Kappa coefficient	0.11	0.55	**0.79**

Bold values indicate the best performance in each category.

From the Table 2, it can be observed that the oriented detection model's performance improves with increasing resolution, reaching its peak performance at a resolution of 512 ×512, where the mean average precision (map) reaches 0.966, mean precision (mp) achieves 0.964, and mean recall (mr) attains 0.962. However, excessively high resolutions (1024 ×1024) result in a decrease in performance, possibly due to overfitting caused by the high resolution. Additionally, as the resolution increases, MC also significantly increases. At a resolution of 1024 ×1024, MC is 1098.68 MB, which is 12.6 times higher than that at a resolution of 256×256(86.69 MB). In comparison, the value of MC at a resolution of 512×512 (290.68 MB) is acceptable and also exhibits the highest model performance.

From the Table 3, the results indicate that reducing the input resolution significantly impacts the scoring system's performance. This performance degradation at lower resolutions is likely due to the loss of fine‐grained details critical for the detection and evaluation of key structures, which are essential for accurate scoring. Therefore, the 512 ×512 resolution is selected as the most optimal choice, offering robust model performance while keeping MC within acceptable limits.

### 4.3 Experiment for PA module

The combination of CNN and RNN has emerged as a widely embraced model for handling spatiotemporal feature data. Within the realm of RNN, there exists a diverse array of architectures, including not only the fundamental RNN networks but also various derivatives such as LSTM,^26^ Bi‐LSTM,^21^ and Gated Recurrent Unit (GRU).^27^ Among these, Bi‐LSTM offers inherent advantages in handling temporal and periodic sequence data, which are highly relevant for echo PA tasks. Bi‐LSTM effectively models bidirectional temporal dependencies, leveraging information from both past and future time steps. This bidirectional nature is particularly advantageous for periodic data, such as cardiac cycles.

This experiment aims to identify the most suitable RNN module for PA of cardiac ultrasound videos, with all models trained and tested on the PA dataset. Each model utilizes a backbone network trained from scratch, and all RNN modules are structured with two layers. Frame error (FE) is used as the evaluation criterion. To ensure the rigor of our experiment, we incorporate 95% confidence intervals and conduct significance analysis to compare the performance of Bi‐LSTM with other RNN models.

For the FE, we conducted normality tests and found that the data do not follow a normal distribution, consequently, we calculated the 95% confidence interval using the median and interquartile range (IQR). Regarding the performance comparison of Bi‐LSTM with other RNN models, we conducted pairwise comparisons using the Wilcoxon signed‐rank test to evaluate statistical significance. Since the FE values (average ED and ES FEs for each video) are continuous and paired across models, the Wilcoxon test was deemed appropriate for this analysis.

The results, presented in Table 4, provide statistical evidence supporting the superior performance of Bi‐LSTM compared to other RNN variants, which confirm our hypothesis. The mean and variance of FE for the ED frames are 1.421±1.261, and for the ES frames are 1.558±1.362, showing significant improvement compared to other modules. This may be related to the periodic characteristics of echo videos, while Bi‐LSTM has strong analytical capabilities for periodic sequences and can capture information from both past and future contexts simultaneously.

**TABLE 4:** Experiment of different RNN networks.

Module	FE of ED		FE of ES	
	μ±σ (95%CI)	*P* value	μ±σ (95%CI)	*P*value
RNN	2.475±2.156[0.0, 8.7]	0.0002	2.341±1.587[0.0, 6.0]	0.0050
GRU	2.173±2.195[0.0, 8.0]	0.0894	2.148±1.473[0.0, 5.25]	0.0159
LSTM	2.143±2.037[0.0, 6.0]	0.0167	2.421±2.316[0.0, 6.25]	0.0030
Bi‐LSTM	**1.421** ± **1.261 [0.0, 4.0]**	**–**	**1.558** ± **1.362 [0.0, 5.0]**	**–**

*Note*: **“–”** indicates the Bi‐LSTM benchmark.

Bold values indicate the best performance in each category.

Abbreviations: Bi‐LSTM, bidirectional long short‐term memory; FE, frame error; GRU, gated recurrent unit; LSTM, long short‐term memory; RNN, recurrent neural network.

### 4.4 Experiments for transfer learning

In our transfer learning experiments, we explored two approaches for selecting a backbone for a multitask model comprising three distinct branches: a PA module, a structural analysis module, and a GC module. In the first approach, we independently trained the object detection module and then utilized its backbone as a shared backbone. Subsequently, we fixed the parameters of this backbone and trained the PA and GC modules. Alternatively, in the second approach, we independently trained the PA module and then used its backbone as a shared backbone. After freezing its parameters, we employed this backbone to train the structural analysis and GC modules. Throughout the experiments, training, and testing data for the three modules were obtained from the PA dataset, AS dataset, and GS dataset, all with a resolution of 512 ×512.

After conducting experiments, we compiled the findings into Table 5. In the table, we observed that utilizing the backbone obtained from the first approach as the shared backbone had minimal impact on the PA module, while the performance of the other two modules remained excellent. However, employing the backbone obtained from the second approach as the shared backbone significantly affected the structure analysis module, with the map dropping to 0.693 compared to 0.962 obtained from the first approach, which is unacceptable. As for the GC module, the impact of both methods was minimal. This phenomenon may be attributed to the low requirement for detailed features in the PA and GC modules, while the structure analysis module demands detailed features. Therefore, it became evident that utilizing the backbone obtained from the first approach led to optimal performance across all modules.

**TABLE 5:** Model performance utilizing backbones trained from different modules as the shared backbone.

Module		Performance		Module		Performance	
		FE of ED (μ±σ)	FE of ED (μ±σ)			FE of ED (μ±σ)	FE of ED (μ±σ)
S‐Phase analysis		1.603 ±1.181	1.681±1.332	P‐phase analysis		**1.421** ± **1.261**	**1.558** ± **1.362**
	MAP	MP	MR		MAP	MP	MR
S‐Structure analysis	**0.966**	**0.964**	**0.962**	P‐structure analysis	0.693	0.670	0.705
	Accuracy	Precision	Recall		Accuracy	Precision	Recall
S‐Gain Classification	**0.992**	**0.992**	**0.993**	P‐gain classification	0.991	0.992	0.992

*Note*: “P‐” indicates the use of the backbone trained from the PA module as the shared backbone. “S‐” indicates the use of the backbone trained from the structure analysis module as the shared backbone.

Bold values indicate the best performance in each category.

Abbreviations: FE, frame error; MP, mean precision; MR, mean recall; PA, phase analysis.

### 4.5 Model performance

**FIGURE 9:**
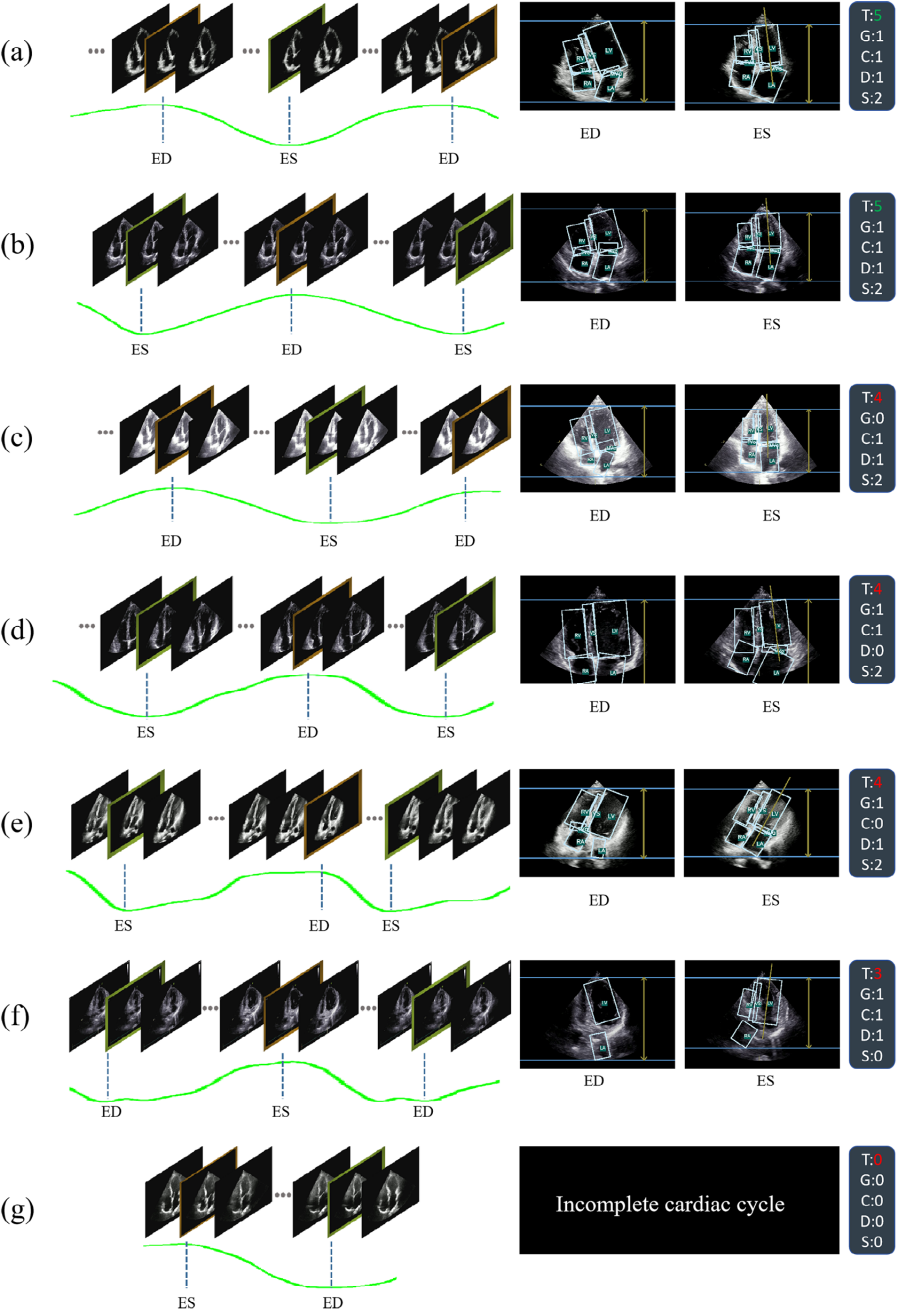
Some examples of overall video scoring. The green curve represents the LVVC regressed by the PA module. The right box represents comprehensive scores corresponding to the criteria in Table 1. T = total score, G = gain, C = CAA, D = depth, S = structure. Note that all the above scores are the same as the GT. CAA, cardiac axis angle; GT, ground truth; LVVC, left ventricular volume curve; PA, phase analysis.

**Overall performance**: The final performance of the entire model is illustrated in Table 4. In the PA module, the FE for ED is 1.603±1.181 and for ES is 1.681±1.332. For the structural analysis module, the map is 0.96, mp is 0.964, and mr is 0.962. In the GC module, the classification accuracy reaches 0.991, and precision reaches 0.993. On our testing setup (a Windows system using an NVIDIA RTX 3060 GPU and 11th Gen Intel(R) Core (TM) i7‐11700 CPU), the model achieved a frames per second (FPS) of 112.4 for the entire process, demonstrating robust real‐time performance. Based on the output of our QC system, we are able to automatically assign a comprehensive score to each echo video. Examples of the scoring results are shown in Figure 9. For each video, only when it meets every QC criterion (a perfect score of 5) can it be considered a standard echo video, as shown in Figure 9a,b; otherwise, it is deemed nonstandard, for example, structural deficiencies are present in the Figure 9f video, while the Figure 9g video lacks complete cardiac cycle, rendering it diagnostically insignificant. Despite having the same scores, the three videos in Figure 9c–e, each have different drawbacks: Figure 9c has excessively high gain, Figure 9d suffers from overly large gain, and Figure 9e has an incorrect CAA. Our QC system not only provides a comprehensive score for the current video but also details the specific conditions of each criterion. This highlights the flaws of each video, offering efficient guidance for sonographers especially novice doctors.

**FIGURE 10:**
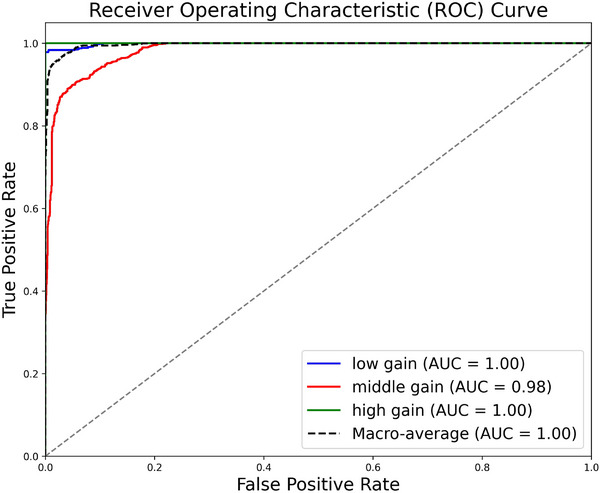
GC perform. GC, gain classification.

**Clinical reliability**: To validate clinical reliability, we randomly gathered a sample of 203 videos. Subsequently, each video in this dataset was individually scored by our automated system and a panel of two experts. We used the scores from the expert panel as GT. Based on these two sets of scores, we evaluated the GC performance and consistency of the model performance:

**1. GC performance**: Figure 10 depicts the receiver operating characteristic (ROC) curve for the GC model across three gain levels: low gain, middle gain, and high gain. The area under the curve (AUC) values demonstrate the model's strong discriminative ability. Specifically, the AUC for low gain and high GCs achieves 1.00, indicating perfect classification performance for these categories. The middle GC achieves an AUC of 0.98, reflecting near‐perfect accuracy with minimal misclassification. The macro‐average AUC of 1.00 further validates the overall robustness and reliability of the model. These results highlight the model's exceptional performance in distinguishing gain levels, with high true positive rates and negligible false positives.

**FIGURE 11:**
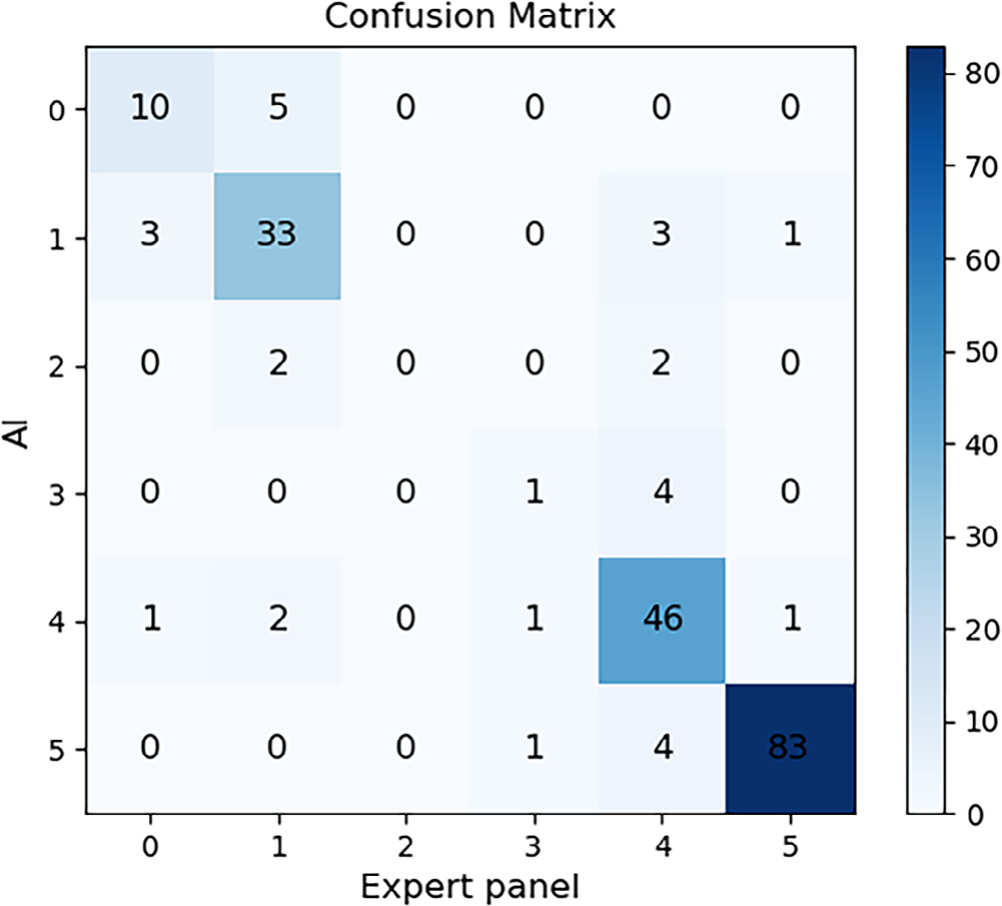
Rows represent the expert panel scores, while columns represent the predicted scores. The numbers in each cell represent the count of samples for the corresponding score.

**2. Consistency**: We conducted a kappa coefficient analysis comparing the scores generated by the expert panel and our model, resulting in a value of 0.79. and the confusion matrix (Figure 11) illustrating the results of scoring the same dataset scores by both AI and expert panel. In Figure 11, our model demonstrates high consistency level overall with experts' ratings. Additional kappa coefficient calculations for nonstandard videos yielded a result of 0.681. Despite a decrease of a certain magnitude compared to the overall value, this outcome remains favorable, considering the intricate nature, and diverse content of nonstandard videos.

Overall, the high kappa coefficient value and the clarity provided by the confusion matrix indicate a significant level of agreement between the automated system and experts, validating the reliability of our automated scoring method. This robust agreement highlights the potential clinical value of our system in reducing doctors' workload and facilitating the rapid acquisition of professional experience for novices.

## 5 DISCUSSION AND CONCLUSION

Automated QC of echo videos is essential in clinical and cardiac ultrasound automation scans. On one hand, it helps alleviate the burden on doctors, on the other hand, it serves as a key step in automated scanning, where standardized views are essential for accurate measurements and diagnoses. In this study, for the first time, we propose a novel multitask system that achieves highly interpretable real‐time QC for echo videos. This system comprises three modules: a PA module for detecting complete cardiac cycles and acquiring QC targets, a structure analysis module for detecting key structures and analyzing depth and CAA, and a GC module for gain analysis. These three modules are integrated on a backbone obtained through transfer learning.

Importantly, we introduce the concept of utilizing a single cardiac cycle as the fundamental QC unit for the first time. This innovative approach standardizes the QC process for echo videos and enables a granular and precise evaluation of each cardiac cycle, which is crucial for detecting anomalies or abnormalities that could impact diagnostic accuracy. In the PA module, we design a simple and efficient LVVC construction function for GT construction in model training, while in the structure analysis module, we utilize postprocessing techniques to efficiently utilize the information inferred by the detection network, thereby enhancing the model's simplicity. Through these three modules, we derive a comprehensive quality score for every cardiac cycle in the video, accompanied by specific improvement insight. Our system demonstrates high performance across all modules, as depicted in Table 4. Additionally, it exhibits strong consistency (with a kappa coefficient of 0.79) in the consistency test conducted with an expert panel, illustrating its robust potential for clinical utility.

Due to the lack of data from other echo views, the current system is only applicable to the A4C view. However, its methodology can be easily extended to other echo views. As mentioned in the introduction, the standard QC processing of echo videos includes assessing the integrity of cardiac cycles, analyzing gain, depth, and CAA, and detecting key structures, with slight variations in QC criteria across different views. With the accumulation of additional data from various echo perspectives, future iterations of the system have the potential to encompass a broader range of views, thereby enhancing its applicability and utility in clinical settings.

In addition to the limitation previously mentioned, another drawback of our scoring system is its inability to capture nuanced changes. Since the scores are discrete, they may not reflect subtle variations in echo images and fail to provide gradual feedback when doctors adjust the probe during scanning. To address the limitations of the current discrete scoring system, we propose two directions for introducing continuous scoring mechanisms. One approach involves employing regression models to evaluate video quality, which would require refining the annotation process. This could involve having experts assign continuous scores during GT labeling or aggregating scores from multiple experts to obtain averaged continuous values. Additionally, leveraging the existing framework, continuous scores can be derived using mathematical and statistical methods. For instance, the confidence score from the detection box could represent the structural quality score, with higher confidence reflecting better quality. Similarly, gain probability in the model output could serve as a representation of quality. For metrics like axes angle or depth, a statistical approach could be adopted by analyzing the GT data distribution, with the likelihood of the model‐predicted values within this distribution acting as a scoring function.

In conclusion, our study underscores the critical role of automated QC in echo video analysis, particularly within clinical and cardiac ultrasound automation scans. Our proposed multitask system represents a significant advancement in this field, providing highly interpretable real‐time QC for echo videos through the integration of three distinct modules: PA, structure analysis, and GC. Notably, we introduce the innovative notion of employing a single cardiac cycle as the foundational QC unit, streamlining the QC procedure of echo video and facilitating meticulous assessment essential for identifying anomalies that may influence diagnostic accuracy.

## CONFLICT OF INTEREST STATEMENT

The authors declare no conflicts of interest.
